# A Study on Thermally Fatigued Phase Transformation and Bending Fracture Mechanisms of 310S Stainless Steel

**DOI:** 10.3390/ma18112654

**Published:** 2025-06-05

**Authors:** Ying-Ting Huang, Yu-Wei Yen, Fei-Yi Hung

**Affiliations:** Department of Materials Science and Engineering, National Cheng Kung University, Tainan 701401, Taiwan

**Keywords:** 310S, thermal cycling, tensile properties, bending, TEM

## Abstract

This study investigates the microstructural evolution and mechanical degradation mechanisms of cold-drawn 310S stainless steel subjected to repeated thermal cycling between 900 °C and room temperature. The results reveal that thermal cycling induces significant lattice distortion, dislocation accumulation, and recrystallization, leading to grain refinement and increased tensile strength. However, these microstructural changes also initiate subsurface cracks and reduce ductility. TGA analysis confirms thermal weight loss from decarburization, especially under oxidative atmospheres. EPMA analysis and tensile tests after thermal cycling reveal that surface cracks formed during thermal cycling act as origins for transgranular crack propagation under tensile stress, significantly reducing fracture resistance. Bending fatigue tests further demonstrate that thermally fatigued specimens exhibit inferior fatigue life compared to raw material, confirming the deteriorating mechanical properties of 310S stainless steel after thermal cycling. Overall, the combined effects of thermal and mechanical fatigue degrade the structural integrity of 310S stainless steel, revealing that lattice distortion and subsurface cracking are the key factors in its embrittlement and reduced fatigue performance.

## 1. Introduction

Austenitic stainless steels with high Ni and Cr content are the 310S stainless steels in the American Iron and Steel Institute (AISI) nomenclature system. Such composition makes 310S stainless steel a heat-resistant type of stainless steel. It is mainly used in high-temperature environments, possesses excellent mechanical properties, and shows high resistance to oxidation and corrosion [1,2]. Therefore, it is widely applied in furnace tubes or conveyor belts in heat treatment equipment, reaction furnaces or distillers in the chemical industry, gas boilers and flue systems in power engineering, and ovens in the food processing industry [3,4,5].

Despite its widespread application, the performance of 310S stainless steel in high-temperature environments is significantly influenced by precipitation behavior. In particular, the formation of Cr-rich σ phase and M23C6 carbides at temperatures ranging from 500 °C to 950 °C can lead to embrittlement and reduced corrosion resistance. Studies suggest that the formation of M23C6 carbides facilitates σ phase precipitation, which compromises the mechanical integrity and service life of stainless steel [6,7]. Previous research has investigated these mechanisms using traditional prolonged isothermal or cyclic aging methods, focusing on the impact of various precipitates on the mechanical properties of 310S stainless steel in high-temperature environments.

Another reason for deterioration is the formation of surface substances under various circumstances [8]. Research in this area explores the behavior of austenitic stainless steel under various environmental conditions, including atmospheric conditions, moisture-rich environments, and extreme settings such as supercritical water or supercritical carbon dioxide. Recent studies have shown that high-temperature oxidation and complex corrosive atmospheres can significantly alter the surface composition and structure of 310S stainless steel, leading to selective oxidation and volatile oxide formation [9,10]. These studies focus on the formation of surface substances, including metal oxides, and their mechanisms, aiming to simulate real-world operational scenarios of 310S stainless steel. By correlating surface elemental analyses with the formation of these substances, the studies provide insights into the consumption of key elements from the parent material and its subsequent impact on the deterioration of 310S stainless steel.

For 3XX stainless steels, reliability assessment mainly relies on the extent of loss in their ductility, as indicated by previous studies [11,12,13]. Under cold working conditions, 310S stainless steel initiates slip systems within the matrix when subjected to an instantaneous external stress, leading to partial lattice shear within the matrix and the formation of twin boundaries in grains. Additionally, these shear behaviors can result in stress-induced martensitic phase transformation, leading to the phenomenon of embrittlement in the mechanical properties of 3XX stainless steels [14,15]. Researchers also focus on changes in microstructure and mechanical properties caused by cold working or investigate the deterioration mechanism of 310S stainless steel through mechanical fatigue. A typical research direction involves utilizing the classic Griffith Law to evaluate the empirical formula for the crack propagation rate under stress in materials and defining the crack propagation rate of stainless steel to assess its service life under different mechanical operating environments [16]. The aforementioned research can simulate the performance of stainless steel in various applications and establish the deterioration mechanism of 310S stainless steel.

Beyond cold work-induced degradation, differences in thermal stability among stainless steels are also critical for understanding material performance under service conditions. Recent studies across various stainless steel grades further emphasize how different heat treatments influence their microstructural evolution and mechanical reliability. Martensitic stainless steels, such as AISI 4140, are typically strengthened through quenching and tempering. However, they tend to exhibit progressive softening under cyclic loading due to the instability of the fine martensitic microstructure [17]. Similarly, the performance of duplex stainless steels is highly sensitive to thermal treatment parameters [18,19]. Insufficient control of heat treatment parameters may lead to the precipitation of brittle intermetallic phases, thereby compromising both toughness and corrosion resistance. In contrast, 310S stainless steel, due to its high Cr and Ni content, exhibits greater thermal stability and retains a single-phase austenitic structure even after thermal cycling.

In high-temperature applications, stainless steel conveyor belts, particularly those made of 310S stainless steel, are widely used due to their excellent mechanical properties and corrosion resistance. However, 310S stainless steel is susceptible to embrittlement and damage when exposed to elevated temperatures [20], especially in environments involving repeated bending and thermal cycling. Among these, bending and fixed strain tensile tests are critical in evaluating its performance under operational conditions. Bending tests simulate the cyclic stress encountered during conveyor operation, while fixed strain tensile tests provide insights into the material’s behavior under constant strain at high temperatures, helping to reveal crack initiation and propagation mechanisms.

Although previous studies have extensively examined the high-temperature characteristics of stainless steel, research focusing on short holding times and thermal cycling applications remains limited [21,22]. This study provides a unique perspective by investigating the effect of short-term thermal cycling on crack propagation under subsequent tensile tests and bending fatigue tests [23,24,25,26]. By integrating thermogravimetric analysis, subsurface EPMA mapping, and TEM-based lattice distortion characterization, this work provides a more comprehensive understanding of the deterioration mechanisms relevant to thermal cyclic environments commonly encountered in industrial applications such as high-temperature conveyor belts.

## 2. Experimental Procedures

**Figure 1:**
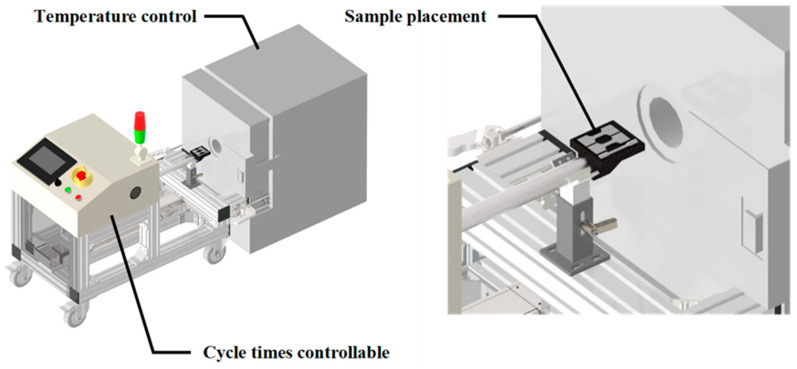
Thermal cycling fatigue equipment schematic [20].

In this study, 310S stainless steel wires with a diameter of 2 mm were selected as raw materials, labeled as RM. The chemical composition of this material is provided in Table 1 (Walsin Lihwa Co., Ltd., Taipei, Taiwan). The thermal fatigue specimens were prepared using a thermal fatigue test machine (CGTH8-L10-400-BR, TOYO, Tokyo, Japan), depicted in Figure 1, following the conditions: 900 °C (holding for 1 min), air-cooled to room temperature (holding for 1 min), repeated for 100 cycles, and the specimen was denoted as C100. The telescopic rod of the test machine allows for controlling the rate at which specimens enter and exit the high-temperature environment. A thermal gravimetric analyzer (Discovery TGA 55, TA Instruments, New Castle, DE, USA) under different atmospheres was used to investigate possible reasons for the mass loss of stainless steel and short-term oxidation behavior.

**Figure 2:**
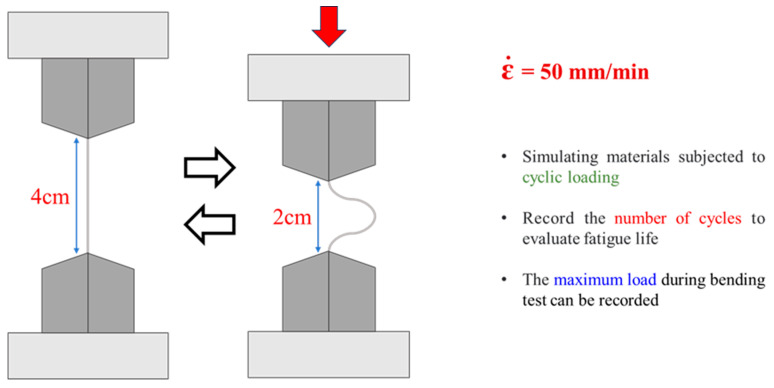
Bend fatigue test schematic.

To establish the deterioration mechanism after thermal cycling, two mechanical tests were conducted. Firstly, C100 specimens were subjected to tensile testing on a universal testing machine (AGX-V2, SHIMADZU Corporation, Kyoto, Japan) to the fixed strain levels of 16% and 32%, with the strain rates set at 8.33 × 10^−3^ s^−1^. Subsequent sub-surface microstructural analysis was performed to confirm the characteristics of thermal cycling cracks and tensile crack growth properties. Secondly, both RM and C100 were subjected to mechanical fatigue tolerance tests using a bending fatigue test machine (EJA Vantage, Thwing-Albert, West Berlin, NJ, USA). Figure 2 illustrates the experimental setup for the bending fatigue test employed in this study. The device allows for adjustment of the spacing between fixtures to apply cyclic bending and recovery to the test specimens. The loading was controlled by displacement, with a compression and tension rate ($\dot{\varepsilon }$) set at 50 mm/min. This displacement control was specifically chosen to ensure that the bending deformation was concentrated at the center of the specimen, minimizing unintended stress distribution near the grips. The fatigue test was performed until failure occurred to determine the fatigue life of both RM and C100. Additionally, in order to examine the early-stage microstructural response to bending, the number of cycles was fixed at 20 for a separate set of samples, labeled RM-B20 and C100-B20, and their degradation was subsequently analyzed. RM, C100, RM-B20, and C100-B20 were subjected to grinding and polishing for microstructural analysis using a metallographic microscope (VHX-7000N, KEYENCE, Osaka, Japan).

XRD analysis (ARL EQUINOX 100 X, Thermal Scientific, Waltham, MA, USA) was conducted to analyze the phase composition in the 2θ range of 20° to 95°. Electron microprobe analysis (JXA-8530F Field Emission Electron Probe Microanalyzer, JEOL, Tokyo, Japan) and wavelength dispersive X-ray spectroscopy (JXA-8900R Electron Probe X-ray Microanalyzer, JEOL) were employed to analyze the distribution of elements in the matrix and sub-surface.

Differences in mechanical properties were evaluated using a Vickers hardness testing instrument (HM-101, Mitutoyo, Kawasaki, Japan) with a load of 0.1 kgf and holding time of 1 s; a tensile testing machine (AGX-V2, SHIMADZU Corporation, Japan) was used to conduct the tensile test, and the strain rate was 1 × 10^−3^ s^−1^. The most noticeable areas of heating and cooling for C100 specimens were near the surface of the specimens, and to avoid intrusion of oxygen due to surface crack generation, the scanning electron microscope system was used to select a 10 μm depth near the surface of C100 for TEM observation. Each set of data is the average of 5–7 test values.

The selected area was etched down using a focused ion beam (Hydra Bio Plasma-FIB, Thermo Fisher, Waltham, MA, USA), fixed on a C-type ring, and further thinned using the ion beam until TEM specimens were obtained. The C100 specimens etched using the FIB system were analyzed using a transmission electron microscope (JEM-2100F CS STEM, JEOL, Japan). The analysis included Mapping, SAED, and HR.

## 3. Results and Discussion

**Figure 3:**
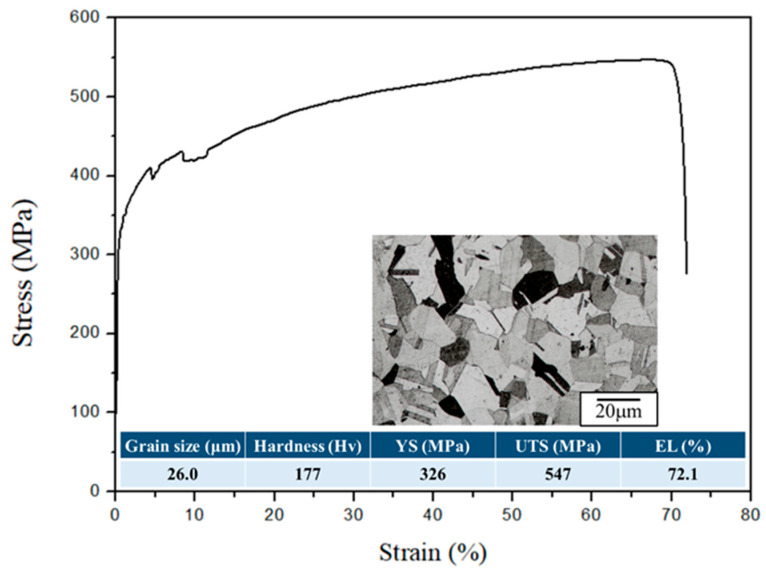
Stress–strain curve and microstructure of raw material (RM).

**Figure 4:**
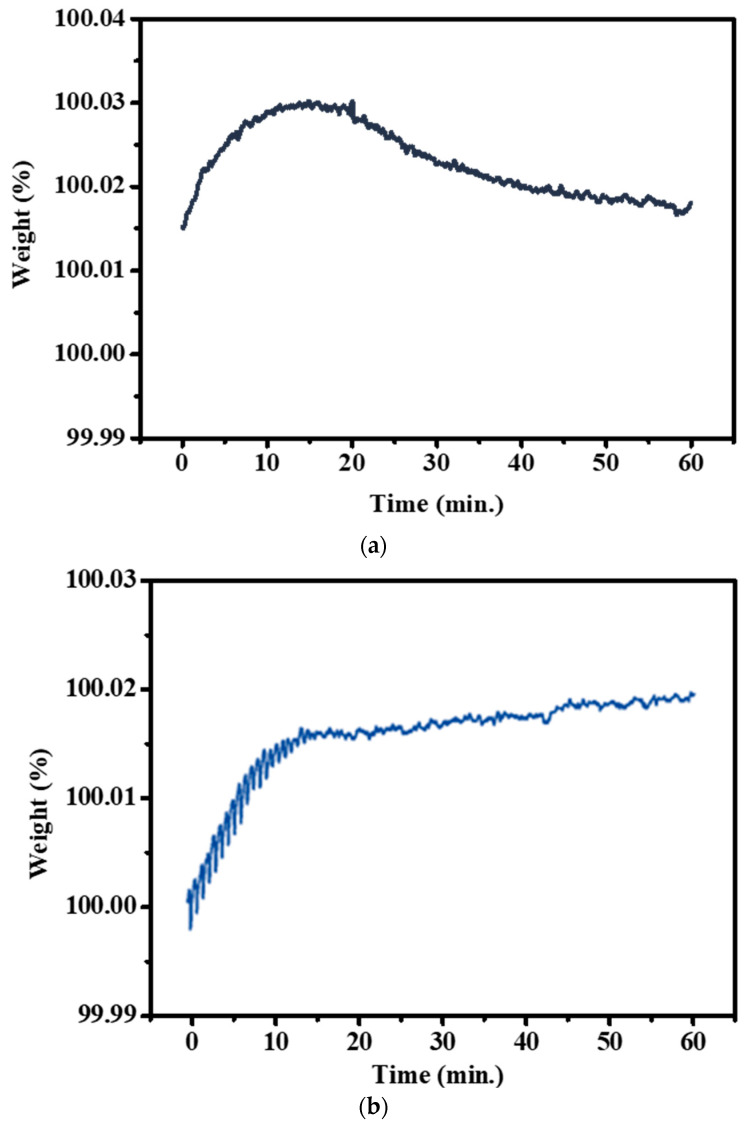
TGA analysis: 900 °C—holding time 1 h. (**a**) Atmospheric atmosphere, (**b**) Ar atmosphere.

The microstructure and the tensile properties of the raw material are depicted in Figure 3, with a hardness of approximately Hv177 and an average grain size of about 26.2 μm. TGA analysis was conducted for 1 h to investigate the initial oxidation behavior of 310S stainless steel. Considering that low-carbon alloy steels are prone to decarburization in high-temperature environments and atmospheric conditions with a higher O_2_ partial pressure, an Ar atmosphere was also established to reduce the gas partial pressure and slow down the rate of decarburization. According to the thermal weight loss analysis results (Figure 4), it was observed that under the condition of holding at 900 °C for 1 h, the weight change was most significant in the first 10 min. After reaching equilibrium, 310S stainless steel continued to undergo decarburization, reflected in the downward trend of the thermal weight loss curve. Compared to the atmospheric atmosphere, 310S stainless steel in an Ar atmosphere still showed weight increase due to oxidation behavior in the first 10 min, as complete oxygen exclusion cannot be achieved inside the TGA instrument. However, after 10 min, the higher O_2_ partial pressure in the atmospheric atmosphere made it difficult for C elements to react and form volatile gases. This resulted in a slower increase in the thermal weight loss curve, indicating that the Ar atmosphere provides protection for stainless steel at high temperatures. Simultaneously, decarburization reactions occurred during the 1 h holding time.

**Figure 5:**
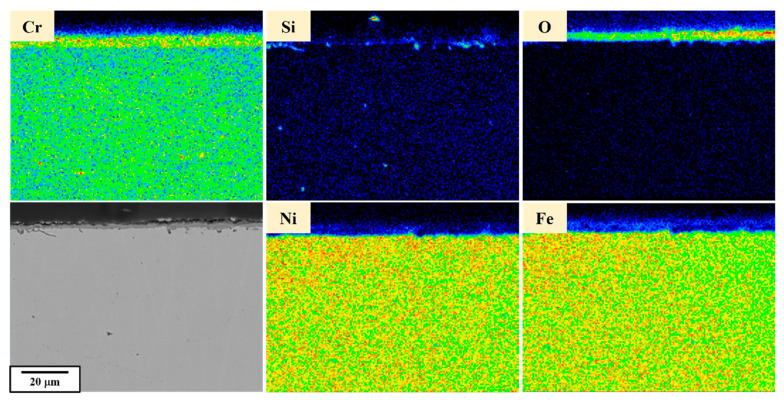
EPMA elemental analysis of raw materials (RM).

SEM and EPMA analyses were conducted on the sub-surface of the raw material as a control group, as shown in Figure 5. The sub-surface matrix exhibited a uniform atomic distribution without significant precipitates, while an oxide layer formed on the surface. From the above results, it can be observed that the raw material exhibits a tendency toward a uniform microstructure and elemental distribution, with no surface cracks.

**Figure 6:**
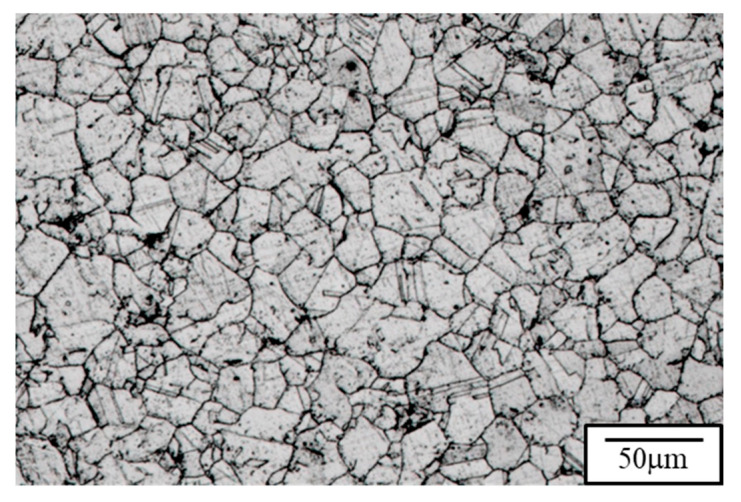
Microstructure of thermal fatigue specimen C100 (grain size: 20.6 μm; hardness: hv 343).

After subjecting the material to 100 cycles of temperature cycling between 900 °C (holding for 1 min) and air cooling to room temperature (holding for 1 min), as depicted in Figure 6, the microstructure exhibits significant changes, primarily characterized by the formation of subgrains due to cyclic thermal stress. While the overall grain size remains mostly unchanged, certain localized regions display smaller grains, suggesting the early stages of recrystallization. This is likely due to the intense temperature fluctuations during the cycling, leading to the continuous generation of defects within the crystal lattice. These defects act as nucleation points for grain recrystallization and refinement. The changes in microstructure are also reflected in the variations in mechanical properties. Investigation of the fatigue material microhardness reveals a significant increase in hardness after the material undergoes severe temperature cycling, consistent with the Hall–Petch relation. The changes in microstructure are also reflected in the variations in tensile properties.

**Figure 7:**
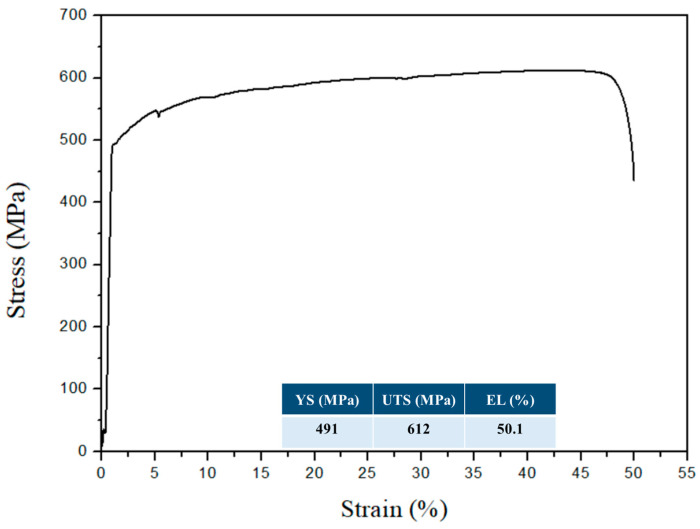
Tensile stress–strain curve of C100.

The tensile properties of C100 are shown in Figure 7, where an increase in tensile strength is observed after the thermal fatigue test, primarily due to grain refinement. The thermal expansion and contraction caused shear-induced defects in the lattice, leading to grain recrystallization and refinement, thereby increasing the tensile strength. However, the accompanying reduction in ductility after thermal cycling is consistent with findings reported in previous studies on 310S and other high-temperature stainless steels [27,28,29]. In particular, the grain boundary carbide precipitation and surface embrittlement observed during long-term isothermal aging in these studies are in agreement with the surface crack formation noted in our C100 specimens.

**Figure 8:**
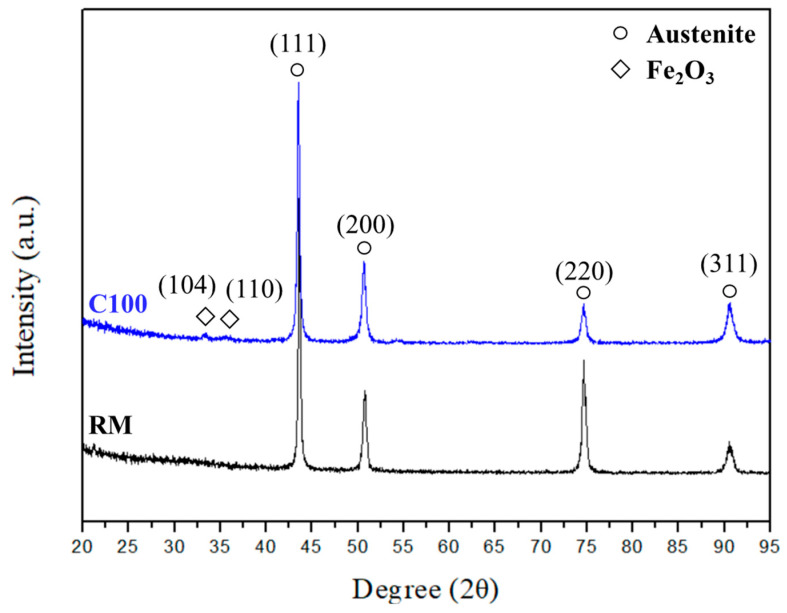
XRD analysis of RM and C100.

Considering the significant changes in mechanical properties, further analysis of the composition of the raw material and C100 was conducted via XRD analysis to investigate whether the steel was affected by structural effects. The results, as shown in Figure 8, reveal a remarkably high similarity between the XRD diffraction patterns of the raw material and C100. Both exhibit γ-iron peaks, indicating that this condition does not induce phase transformation in 310S. Therefore, the increase in tensile strength of 310S after thermal fatigue can be attributed to the defect-induced grain recrystallization resulting from thermal expansion and contraction.

**Figure 9:**
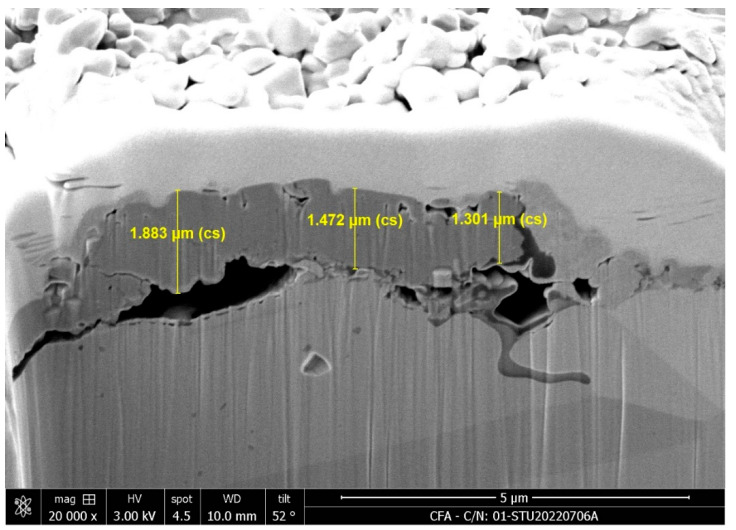
FIB image of C100 cross-section.

**Figure 10:**
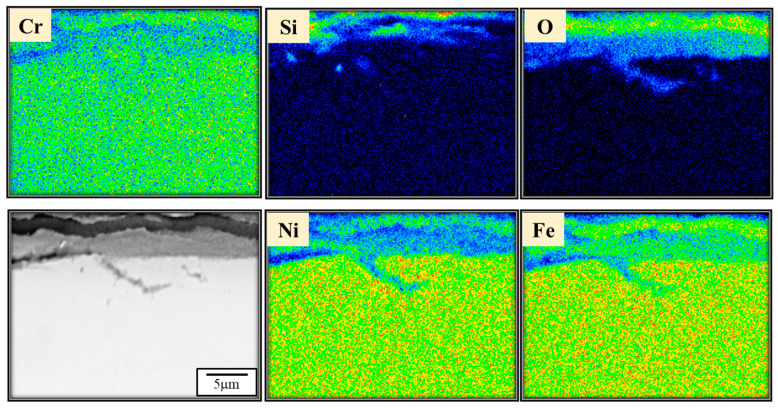
EPMA elemental analysis of near-surface cracks in C100.

However, solely from the perspective of grain refinement, it might be challenging to explain the observed decrease in ductility of 310S after the thermal fatigue test. Additionally, considering that 310S stainless steel may develop cracks or experience element segregation due to uneven heating during cyclic temperature fluctuations, leading to possible crack initiation, FIB and EPMA analyses were performed on the sub-surface of C100. From Figure 9, the FIB image reveals a continuous oxidation film on the surface with a thickness ranging from approximately 1.3 μm to 1.9 μm. Beneath the oxidation layer, cracks are visibly propagating into the subsurface region, suggesting structural degradation due to thermal fatigue. Additionally, the EPMA results shown in Figure 10 further support these observations, highlighting the accumulation of silicon near the surface, which may contribute to the embrittlement and crack formation observed in the FIB image. Considering the possibility of crack generation after the thermal fatigue test, it is reasonable to assume that these cracks may affect the ductility of the material.

**Figure 11:**
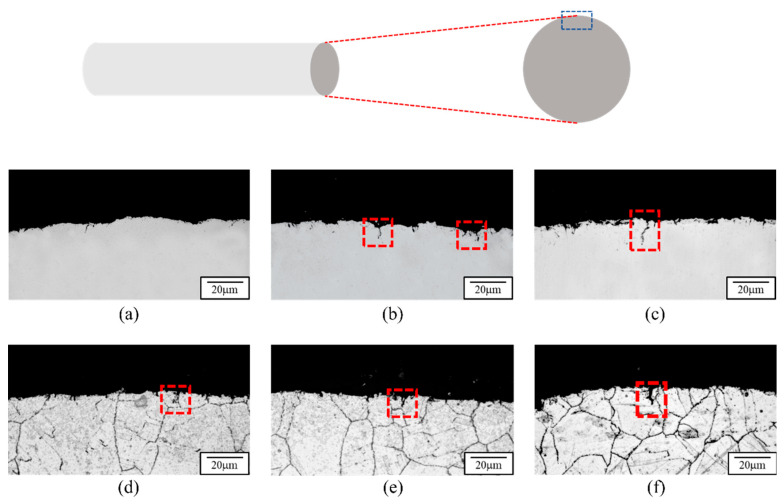
Crack Propagation in 310S Stainless Steel After Thermal Fatigue and Tensile Strain. (**a**) Surface condition after thermal fatigue (100 cycles). (**b**) Thermal fatigue (100 cycles) followed by tensile strain of 16%. (**c**) Thermal fatigue (100 cycles) followed by tensile strain of 32%. (**d**–**f**) Corresponding microstructures with etching, showing crack propagation for (**a**), (**b**), and (**c**), respectively.

Considering that 310S stainless steel, used in this study, is mainly employed in high-temperature conveyor belts that may experience relaxation and subsequent re-tightening during actual operation, it is subjected to external mechanical forces. Therefore, after undergoing thermal fatigue testing, tensile testing was conducted. The elongation was set to 1/3 and 2/3 of the maximum elongation capacity of C100 (approximately 16% and 32%). The microstructural changes in C100 during tensile testing are illustrated in Figure 11. From Figure 11, it can be observed that after experiencing elongations of 16% and 32%, the near-surface cracks in C100 exhibit significant propagation due to tension. At 32% elongation, the depth of the cracks has reached approximately 4–5 times that of C100 material. Therefore, it is inferred that the primary reason for the decreased ductility of 310S stainless steel after thermal fatigue testing is likely due to crack propagation.

Further investigation into the relationship between cracks and grain boundaries, as shown in Figure 11d–f, reveals that the majority of cracks in 310S stainless steel, after undergoing thermal fatigue followed by tension, propagate transgranularly toward the center of the 310S stainless steel. This characteristic is typical of brittle materials and confirms that 310S stainless steel indeed experiences embrittlement after thermal fatigue. This implies that as the strain increases, there is an accelerating trend in crack propagation for the fatigued material. Therefore, it is inferred that in higher strain ranges, cracks dominate the mechanical property effects of the fatigued material more significantly. Consequently, when comparing the ductility of RM and C100 with and without initial cracks, there is a significant discrepancy between the two.

**Figure 12:**
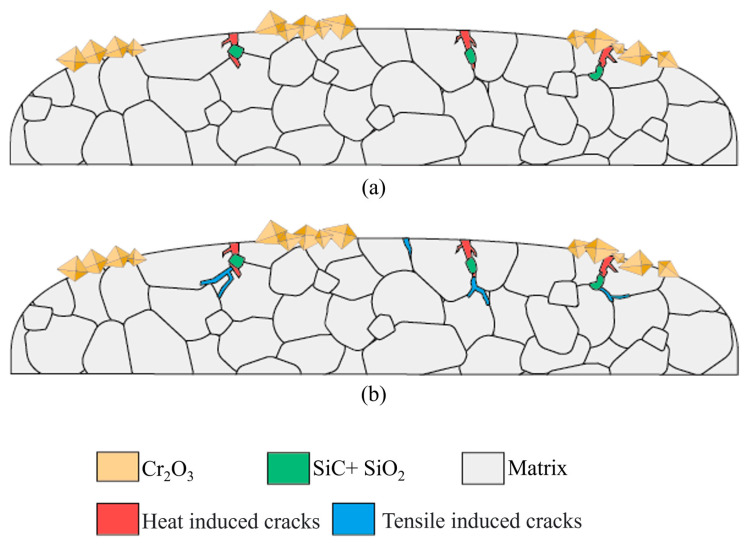
Mechanism of crack propagation after thermal fatigue. (**a**) Initial crack formation after thermal fatigue. (**b**) Propagation of tensile strain-induced cracks.

The mechanisms of subsurface initial cracks and their propagation are illustrated in Figure 12. Figure 12a shows the element distribution and crack characteristics of 310S stainless steel after thermal fatigue testing. Due to the severe thermal expansion and contraction cycles near the surface caused by the thermal fatigue test, after 100 fatigue cycles, cracks with an average initial depth of about 5 μm are generated. Subsequent tensile stress after thermal fatigue leads to further propagation of these cracks, as shown in Figure 12b. The crack propagation rate increases with the amount of elongation, resulting in a decrease in the ductility of 310S stainless steel.

**Figure 13:**
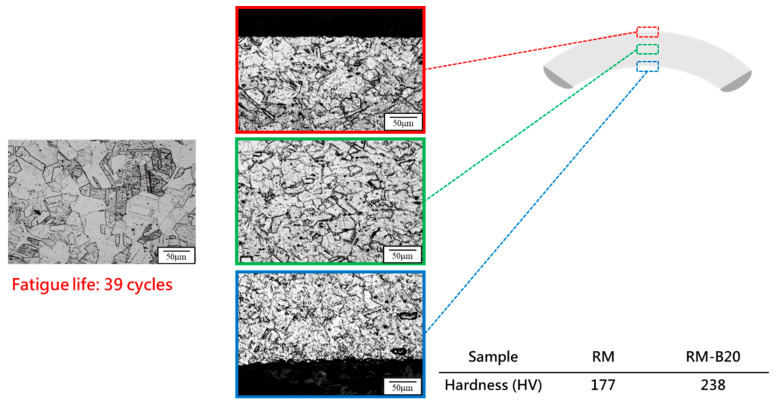
Microstructural characteristics of RM before and after bending: (Before bending: HV177; After bending: HV238; Fatigue life: 39 cycles).

**Figure 14:**
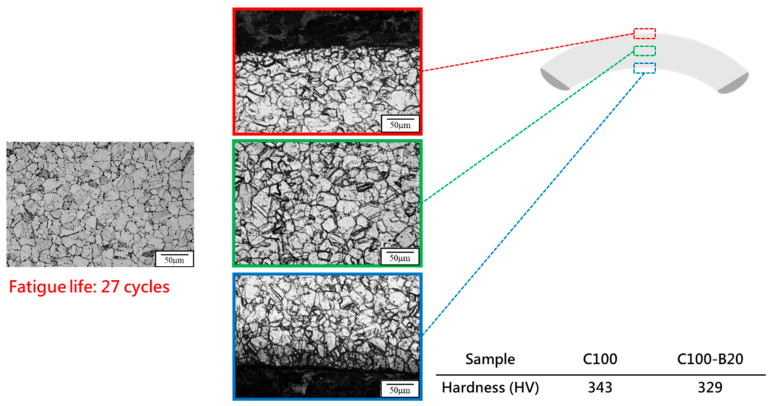
Microstructural characteristics of C100 before and after bending. (Before bending: HV343; After bending: HV329; Fatigue life: 27 cycles).

To integrate both thermal and mechanical fatigue effects on 310S stainless steel and simulate service conditions in woven high-temperature conveyor belts, RM was used as a control group. Both RM and C100 were subjected to bending fatigue tests. Microstructural observations from Figure 13 and Figure 14 show that after bending fatigue, RM exhibited clear signs of work hardening, with a significant increase in microhardness. The bending fatigue induced the formation of deformation twins within the material, and cracks were primarily observed in the upper region of the specimens, where tensile stress is concentrated. In contrast, fewer cracks were found in the lower region under compressive stress. Moreover, in the middle region under minimal stress, microstructural features remained relatively well preserved under similar corrosion conditions. This hardening behavior aligns with previously reported twin-induced strengthening in cold-worked austenitic stainless steels [30,31,32]. Conversely, the absence of deformation twins in the thermally fatigued specimens (C100) can be attributed to thermally activated recrystallization, which eliminates dislocation structures and suppresses the conditions required for twin formation.

**Figure 15:**
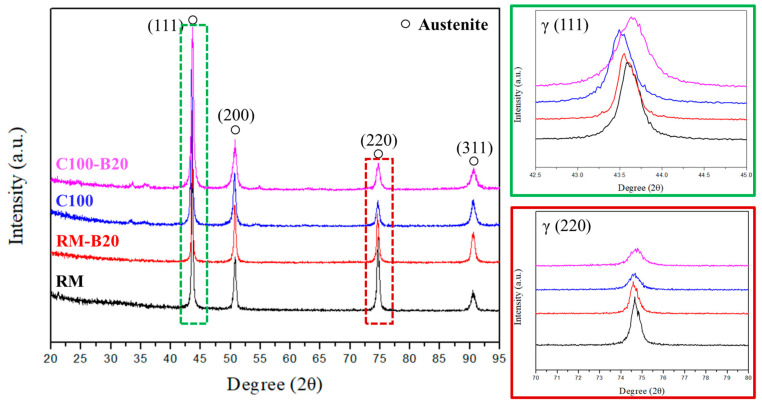
XRD analysis: RM, RM with 20 bending fatigue cycles (RM-B20). C100, C100 with 20 bending fatigue cycles (C100-B20).

XRD analysis results from Figure 15 indicate that without stress-induced phase transformations, the hardening observed in RM mainly stems from mechanical fatigue-induced work hardening. In comparison, while RM exhibited a significant increase in hardness, C100 showed no apparent increase after bending fatigue. This outcome may suggest that the extent of work hardening due to the formation of new defects inside 310S stainless steel after thermal fatigue, further contributing to work hardening, has limitations. This indicates that there is a certain limit to the hardening degree due to defects, and after undergoing thermal fatigue followed by bending tests, the material may have reached this limit. The bending fatigue life of RM and C100 also indirectly corroborates this, with C100, having defects formed internally due to thermal expansion and contraction, showing poorer fatigue life.

The XRD analysis results in Figure 15 show a shift in the main peak (111) after bending tests, indicating changes in the internal lattice constant due to applied external stress. Additionally, a peak was observed in the XRD spectrum at 2θ = 32.5–35°, identified as Fe_2_O_3_ and Fe_3_O_4_ after comparison. It is inferred that under short-term thermal fatigue conditions, Fe elements near the surface preferentially form iron oxide. Regardless of thermal or bending fatigue, there were no significant structural differences observed in 310S stainless steel, suggesting that stress-induced phase transformations cannot be observed through XRD analysis under these conditions. While there were no significant changes in peak width and position before and after bending fatigue tests for both RM and C100, the peak shift was more apparent in C100. This suggests that defects formed internally due to thermal expansion and contraction in 310S stainless steel after thermal fatigue, combined with newly formed defects from cold working, create a larger stress field, leading to a more pronounced effect on lattice deformation and peak shifts in XRD.

**Figure 16:**
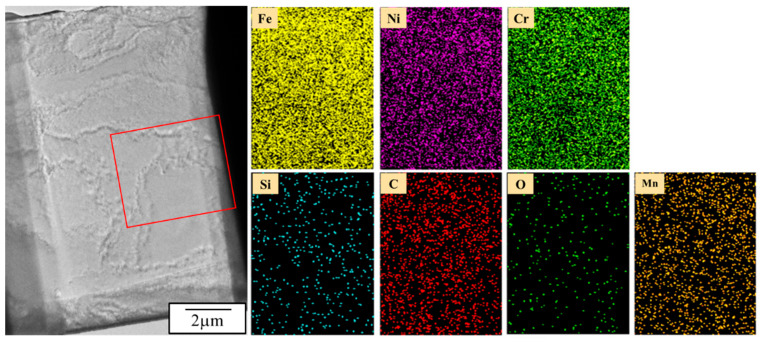
TEM mapping analysis of C100.

**Figure 17:**
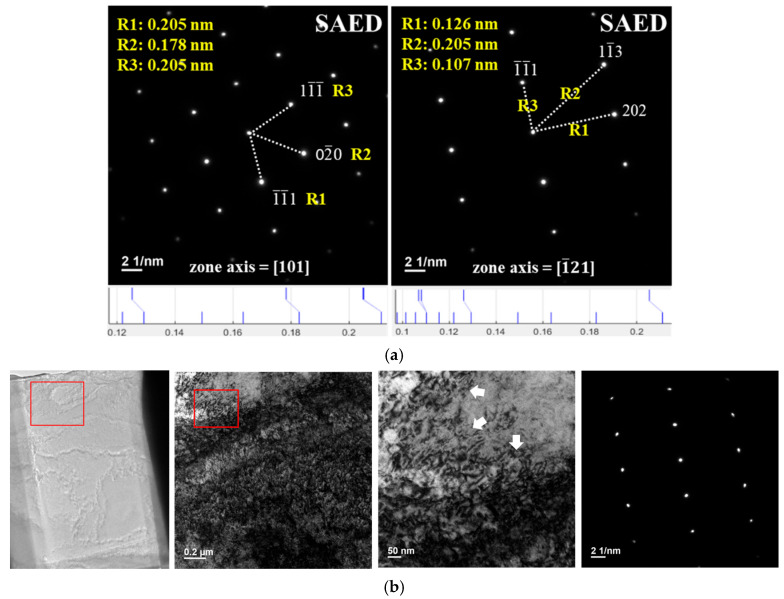
TEM analysis of C100. (**a**) SAED analysis. (**b**) Dislocation accumulation + diffraction patterns.

Figure 16 presents the results of near-surface TEM analysis. The mapping analysis reveals a uniform distribution of elements within the image range. It indicates that under the condition of 900 °C (holding for 1 min) ⇄ room temperature (holding for 1 min) cycling 100 times, all elements exhibit homogeneous distribution. This is likely due to the insufficient total holding time, preventing atoms from having enough time to move at high temperatures. TEM diffraction analysis, as depicted in Figure 17a, shows that the base phase is the γ-Fe lattice. Combined with the bright-field and SAED analysis in Figure 17b, after 100 cycles, there is no apparent phase transformation near the surface of the 310S stainless steel. However, lattice distortion is observed, reflected in the deformation of diffraction spots. Additionally, intense thermal expansion and contraction led to dislocation clustering, corresponding to the XRD diffraction spectrum results for the C100 specimen. It is inferred that thermal cycling causes lattice distortion, potentially satisfying the conditions for stress-induced phase transformation locally. Consequently, the C100 specimen exhibits embrittlement, with thermal cracking on the steel surface and hardening within the base material due to lattice distortion.

## 4. Conclusions

(1) Thermal fatigue testing subjects 310S stainless steel to cyclic thermal stress, inducing lattice distortion, recrystallization, and localized grain refinement. High-temperature cycling (from 900 °C to room temperature) promotes dislocation accumulation and grain boundary movement, facilitating recrystallization. Additionally, atomic stacking faults formed during thermal cycling contribute to the nucleation of annealing twins, a characteristic feature of recrystallized grains.

(2) Subsurface crack formation and propagation reduce the ductility of 310S stainless steel. These cracks act as stress concentrators, leading to premature failure under loading and a marked reduction in the service life of 310S stainless steel. The combined effects of thermal stress and mechanical fatigue testing exacerbate crack growth, making subsurface cracks a critical factor in the degradation of ductility and structural integrity.

(3) The lattice distortion induced by thermal fatigue testing weakens the microstructure of 310S stainless steel, increasing brittleness and reducing resistance to bending. This facilitates the initiation and propagation of subsurface cracks, which expand further during mechanical testing. The combined effects of lattice distortion and crack propagation synergistically degrade the mechanical properties of the material.

(4) The results provide new insight into the deterioration mechanism of 310S stainless steel under realistic thermal and mechanical service conditions, suggesting that even short-term cyclic thermal exposure can accelerate embrittlement. Thus, more conservative design approaches or protective measures may be necessary in industrial applications such as high-temperature conveyor systems.

## Figures and Tables

**Table 1:** Chemical composition of the as-received AISI 310S stainless steel (wt.%).

C	Cr	Ni	Mn	Si	P	S	Fe
0.080	25.000	21.000	2.000	1.500	0.045	0.030	Bal.

## Data Availability

The raw data supporting the conclusions of this article will be made available by the authors on request.
